# Supplementary material for: Signatures of selection and mechanisms of insecticide resistance in Ugandan Anopheles funestus: Insights from embedding translational genomics into the LLINEUP cluster randomised trial
Source: bioRxiv. 2025 Jun 19:2025.06.17.659961. Preprint. [Version 1] doi: 10.1101/2025.06.17.659961 (PMC12262602; doi:10.1101/2025.06.17.659961)
Supplement: Supplement 1 [file media-1.pdf]

Signatures of selection and mechanisms of insecticide resistance in Ugandan *Anopheles funestus*: Insights from embedding translational genomics into the LLINEUP cluster randomised trial.

Lilian Namuli-Kayondo, Sanjay C Nagi, Harun Njoroge Nganga, Anastasia Hernandez-Koutoucheva, Daniel P. McDermott, Samuel Gonahasa, Amy Lynd, Ambrose Oruni, Catherine Maiteki-Sebuguzi, Jimmy Opigo, Adoke Yeka, Agaba Katureebe, Mary Kyohere, Moses R Kamya, Grant Dorsey, Janet Hemingway, Sarah G Staedke, Samuel L Nsobya, Joaniter I Nankabirwa, Jonathan Kayondo, Chris Clarkson, Alistair Miles, Mara K N Lawniczak, Eric R Lucas & Martin J Donnelly

Lilian Namuli-Kayondo
Liverpool School of Tropical Medicine, Pembroke Place, Liverpool L3 5QA, UK and Infectious Diseases Research Collaboration, 2C Nakasero Hill Road, Kampala, Uganda and National Malaria Elimination Division, Ministry of Health, Kampala, Uganda.
lilian.namuli@lstmed.ac.uk

Sanjay C Nagi*
Liverpool School of Tropical Medicine, Pembroke Place, Liverpool L3 5QA, UK.
sanjay.nagi@lstmed.ac.uk

Harun Njoroge Nganga
Liverpool School of Tropical Medicine, Pembroke Place, Liverpool L3 5QA, UK.
harun-njoroge.nganga@lstmed.ac.uk

Anastasia Hernandez-Koutoucheva
Wellcome Sanger Institute, Hinxton, United Kingdom.
ah32@sanger.ac.uk

Daniel P. McDermott
Liverpool School of Tropical Medicine, Pembroke Place, Liverpool L3 5QA, UK.
daniel.mcdermott@lstmed.ac.uk

Samuel Gonahasa
Infectious Diseases Research Collaboration, 2C Nakasero Hill Road, Kampala, Uganda. and Liverpool School of Tropical Medicine, Pembroke Place, Liverpool L3 5QA, UK.
sgonahasa1983@gmail.com

Amy Lynd
Liverpool School of Tropical Medicine, Pembroke Place, Liverpool L3 5QA, UK.
amy.lynd@lstmed.ac.uk

Ambrose Oruni
Liverpool School of Tropical Medicine, Pembroke Place, Liverpool L3 5QA, UK.
orambrose@yahoo.com

Catherine Maiteki-Sebuguzi
National Malaria Elimination Division, Ministry of Health, Kampala, Uganda
cmaiteki@yahoo.com

Jimmy Opigo
National Malaria Elimination Division, Ministry of Health, Kampala, Uganda
opigojimmy@gmail.com

Adoke Yeka
Infectious Diseases Research Collaboration, 2C Nakasero Hill Road, Kampala, Uganda
yadoke@yahoo.com

Agaba Katureebe
Infectious Diseases Research Collaboration, 2C Nakasero Hill Road, Kampala, Uganda
agabaik@gmail.com

Mary Kyohere
Infectious Diseases Research Collaboration, 2C Nakasero Hill Road, Kampala, Uganda
mkyohere@sgul.ac.uk

Moses R. Kamya
Makerere University College of Health Sciences.
Infectious Diseases Research Collaboration, 2C Nakasero Hill Road, Kampala, Uganda.
mkamya@infocom.co.ug

Grant Dorsey
University of California, San Francisco, San Francisco, CA 94110 USA
grant.dorsey@ucsf.edu

Janet Hemingway
Liverpool School of Tropical Medicine, Pembroke Place, Liverpool L3 5QA, UK.
janet.hemingway@lstmed.ac.uk

Sarah G Staedke
Liverpool School of Tropical Medicine, Pembroke Place, Liverpool L3 5QA, UK.
sarah.staedke@lstmed.ac.uk

Samuel L Nsobya
Infectious Diseases Research Collaboration, 2C Nakasero Hill Road, Kampala, Uganda.
samnsobya@yahoo.co.uk

Joaniter I Nankabirwa
Makerere University College of Health Sciences.
Infectious Diseases Research Collaboration, 2C Nakasero Hill Road, Kampala, Uganda.
jnankabirwa@yahoo.co.uk

Jonathan Kayondo
Uganda Virus Research Institute, Entebbe, Uganda. jkayondo@gmail.com

Chris Clarkson,
Wellcome Sanger Institute, Hinxton, United Kingdom.
cc28@sanger.ac.uk

Alistair Miles,
Wellcome Sanger Institute, Hinxton, United Kingdom.
alistair.miles@sanger.ac.uk

Mara K N Lawniczak
Wellcome Sanger Institute, Hinxton, United Kingdom.
mara@sanger.ac.uk

Eric R Lucas
Liverpool School of Tropical Medicine, Pembroke Place, Liverpool L3 5QA, UK.
eric.lucas@lstmed.ac.uk

Martin J Donnelly*
Liverpool School of Tropical Medicine, Pembroke Place, Liverpool L3 5QA, UK.
martin.donnelly@lstmed.ac.uk

*corresponding authors

Supplementary material

Supplementary Table 1

Intervention arm	Pre-intervention		6 months		12 months		18 months		Post-intervention	
	AF	AG	AF	AG	AF	AG	AF	AG	AF	AG
**Non-PBO**	176	549	149	190	177	384	394	295	242	609
**PBO**	257	816	45	37	73	119	325	78	193	280

Table 1: Total number of PCR confirmed *An. gambiae* s.l. and *An. funestus* s.l. vector densities during the LLINEUP trial. AG= *Anopheles gambiae* s.l.; AF= *Anopheles funestus* s.l.

Supplementary Table 2

	Estimated		Std error		Z value		Pr(>|z|)	
	**AG**	**AF**	**AG**	**AF**	**AG**	**AF**	**AG**	**AF**
**Intercept**	1.860	1.487	0.102	0.133	18.172	11.207	< 2e-16 ***	<2e-16 ***
**6 months**	-0.622	-0.274	0.173	0.186	-3.594	-1.476	0.000326 ***	0.140
**12 months**	-0.468	-0.384	0.135	0.165	-3.461	-2.324	.000539 ***	0.020
**18 months**	-0.209	0.459	0.190	0.154	-1.102	2.981	0.270	0.00287 **
**Post intervention**	-0.116	-0.009	0.126	0.154	-0.921	-0.060	0.357	0.952
**LLIN type**	-0.235	-0.123	0.102	0.108	-2.297	-1.143	0.021611 *	0.253

Table 2: Generalised linear mixed model of *An. gambiae* s.l. and *An. funestus* s.l. vector density during the LLINEUP trial. Results of a nb-model <- glmmTB (species~ round+LLINtype+ (1|HH), family =n.bionm2). AG= *Anopheles gambiae* s.l.; AF= *Anopheles funestus* s.l.; HH= Household; n.bionm2= negative binomial type2, Std error= standard error; ***= p≤ 0.001; **=p ≤ 0.01 and *=p ≤ 0.05

Supplementary Figure 1

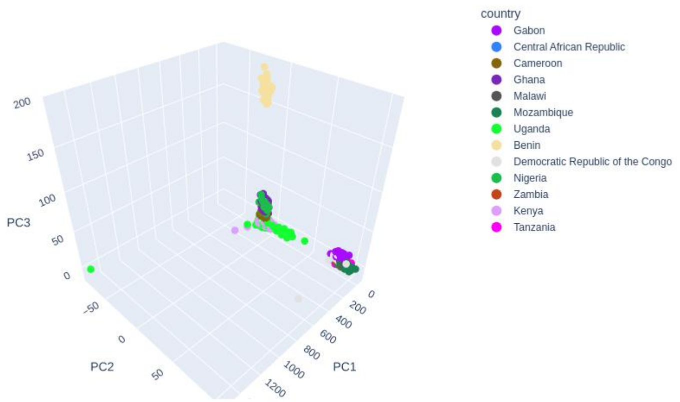

Fig 1: An. funestus Principal component analysis. Using the genomic region 2R:60-80Mbps to avoid any polymorphic chromosomal inversions showing Ugandan LLINEUP An. funestus populations clustering with equatorial populations.

Supplementary Figure 2

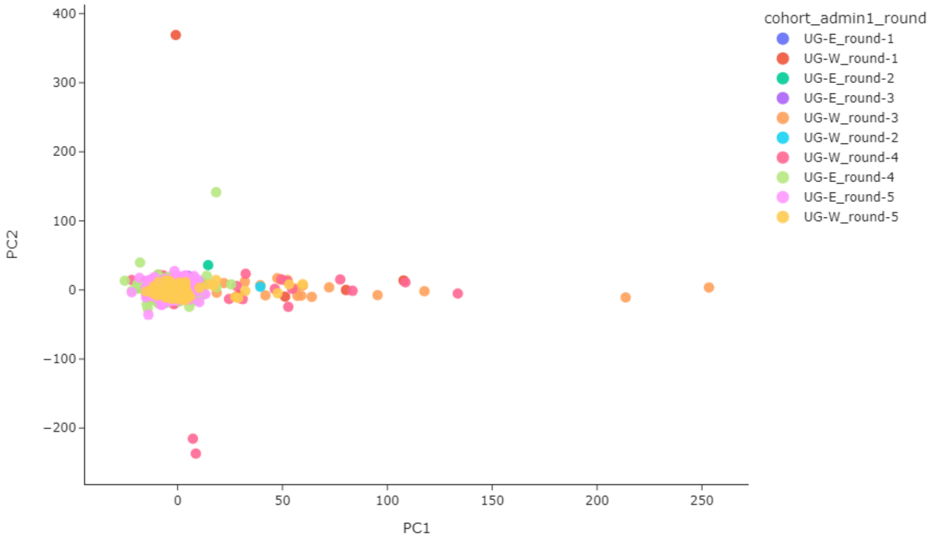

Fig 2: An. funestus Principal component analysis. Using the genomic region 2R:60-80Mbps showing lack of structure within the An. funestus populations. Although geographically separated and collected over different temporal spaces, the East and Western Uganda An. funestus cluster together highlighting the lack of structure. UG-E= Uganda Eastern region, UG-W, Uganda Western region, round-1= Pre-intervention, round-2= 6 months, round-3= 12 months, round-4= 18 months, round-5= post- intervention.

Supplementary Table 3

Region	**Median F_{ST} *(p value)***
Between population	0.00107
Within West	0.00306
Within East	-0.00078
Between population versus within West	411754 (8.2718e-12)
Between population versus within East	598502 (2.3845e-14)

Supplementary table 3: Pairwise F_{ST} within East and Western Uganda versus between East and Western Uganda F_{ST}.

Supplementary Figure 3

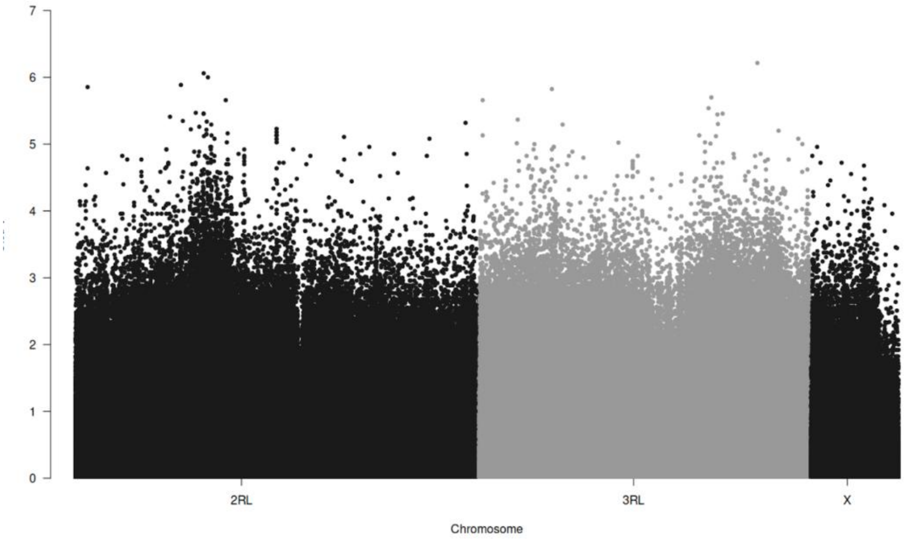

Fig.3: Manhattan plot of SNPs between pre and post intervention An. funestus population. Used a generalised linear mixed model, and no SNP was significantly associated with LLIN use. Tested the association between allele frequencies and intervention (pre-intervention versus post-intervention) and net type as fixed effect, and health subdistricts as random effects.

Supplementary Table 4:

Chromosome	Gene	Peak center	Peak start	Peak stop
**2RL**	*RP1- locus*	8667967	8658455	8683454
	Complexin transcript variant	51660985	51657957	51663230
		57731386	57714091	57740033
	Gste-2	67239558	67236023	67241326
**3RL**		24915154	24855951	24944756
		29245108	29237124	29249101
	TMC-1	67929193	67925771	67931656
**X**	*Cyp9k1*	8452011	8448380	8459274
	Dgk	13639410	13636759	13641768

Table 4: showing peak signal (p=0.05) detection in An. funestus. We identified regions within the genome that were under selection

Supplementary Figure 4

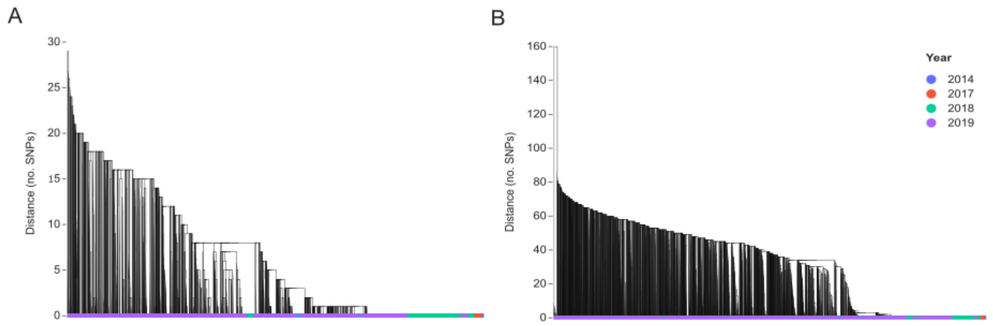

Fig 4: Hierarchical clustering dendrogram of haplotypes *over the TMC-1 (3RL: 67,925,771- 67,931,656- plot A&B) and Dgk (X: 13,590,000-13,690,044- plot C&D). Dendrogram leaves are labeled by year of sample collection (Plot A&B).*

Supplementary Figure 5

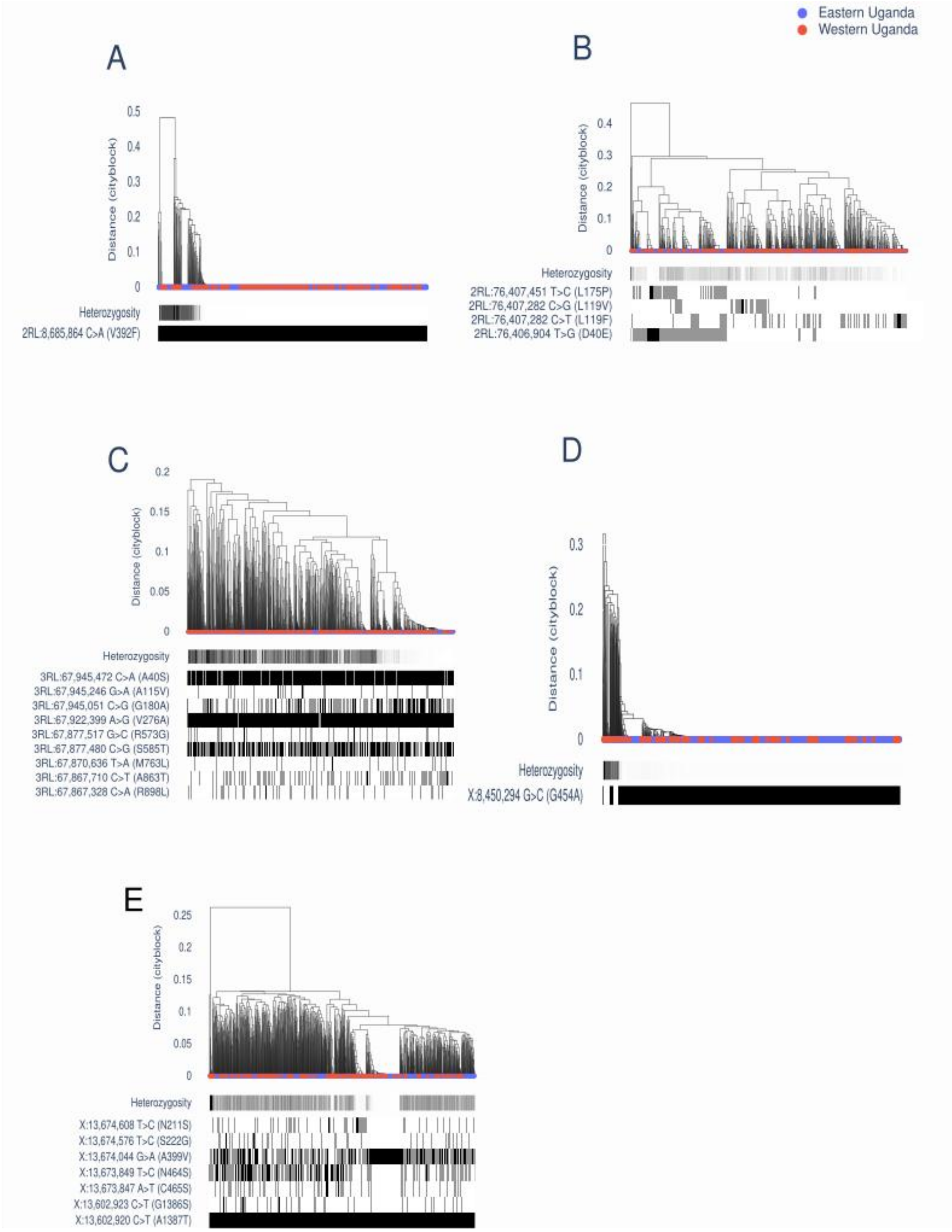

Fig 5: Diplotype clustering: *RP1 locus (2RL: 8,658,455-8,683,454-plot A), B) Gste2 (2RL: 76,406,705-76,407,655-plot B), TMC (3RL: 67,925,771-67,931,656-plot C), Cyp9k1 (X: 8,440,000-8,451,000-plot D) and Dgk (X: 13,590,000-13,690,044-plot E), and loci. Pairwise distance between diplotypes was calculated using genetic distance based on city-block distance and complete linkage plotted as hierarchical clusters. The colouring of the dendrogram leaves corresponds to the geographical*

region from which each individual sample was collected; below the dendrogram is the assigned sample heterozygosity and gene SNP mutations are displayed as horizontal bars.

Supplementary Table 5

**Region**	**Trial arm**	**Pre-intervention**	**6 months**	**12 months**	**18 months**	**Post intervention**
**Eastern**	Non-PBO	3	45	17	230	195
	PBO	5	11	23	203	117
**Western**	Non-PBO	25	1	24	31	19
	PBO	6	1	2	48	39
**Total**		**39**	**58**	**66**	**512**	**370**

Table 5: Number of An. funestus collected from East and Western Uganda in both PBO and Non-PBO arms during the five rounds of the trial.
